# Development of a fast liquid chromatography-tandem mass spectrometry method for simultaneous quantification of neurotransmitters in murine microdialysate

**DOI:** 10.1007/s00216-020-02906-z

**Published:** 2020-09-17

**Authors:** Christin Helmschrodt, Susen Becker, Stefanie Perl, Anja Schulz, Angelika Richter

**Affiliations:** Institute of Pharmacology, Pharmacy and Toxicology, Faculty of Veterinary Medicine, University of Leipzig, An den Tierkliniken 15, 04103 Leipzig, Germany

**Keywords:** Microdialysis, Neurotransmitter, Neurochemistry, LC-MS/MS, Basal values, Murine microdialysate

## Abstract

The continuous measurement of multiple neurotransmitters in microdialysate of freely moving mice to study neurochemical changes in specific brain regions requires a rapid and very sensitive quantitative analytical method. The quantitative analysis of 11 neurotransmitters and metabolites, including serotonin (5-HT), 5-hydroxyindoleacetic acid (5-HIAA), melatonin (ME), dopamine (DA), levodopa (l-DOPA), 3-methoxytyramine (3-MT), norepinephrine (NE), epinephrine (EP), acetylcholine (ACh), choline (Ch), and γ-aminobutyric acid (GABA), was performed using a biphenyl column coupled to an API-QTrap 3200 (AB SCIEX) mass spectrometer in positive electrospray ionization mode. To the microdialysate samples, 0.5 ng of isotopically labeled standard was added for analyte quantification. A rapid liquid chromatography-tandem mass spectrometry (LC-MS/MS) method was developed and validated for the simultaneous analysis of monoamines, their precursor, and metabolites, as well as ACh, Ch, and GABA in murine microdialysate within 7.0 min. The limit of detection in artificial CSF ranged from 0.005 ng/mL (ME) to 0.75 ng/mL (NE and GABA). A comprehensive pre-analytical protocol was validated. Recovery was between 87 and 117% for neurotransmitter concentrations from 0.6 to 45 ng/mL with an inter-day accuracy of below 20%. Basal neurotransmitter values were determined in the striatum of mice over a time period of 3 h. This LC-MS/MS method, including a short and gentle sample preparation, is suitable for simultaneous measurements of neurotransmitters in murine cerebral microdialysate and enables the determination of basal neurotransmitter levels in specific brain regions to detect disease-related and drug-induced neurochemical changes.

Graphical abstract
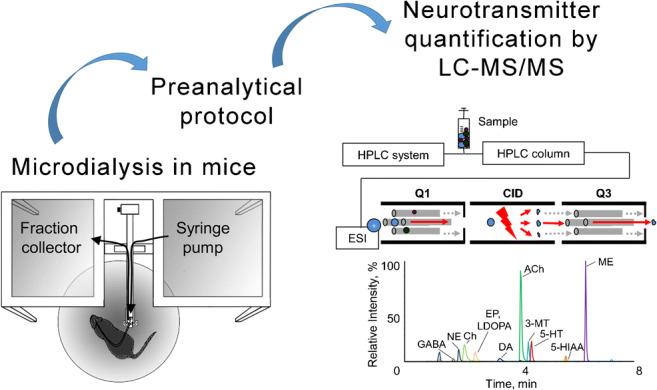

## Introduction

Neurochemistry provides insights into the pathophysiology of neurological diseases and mechanisms of drugs. In addition to anatomical and behavioral methods, this field complements the study of the integrity of neurotransmitter pathways. In vivo microdialysis (MD), as a minimally invasive sampling technique, permits the continuous analysis of extracellular NTs in freely moving animals by monitoring changes in the extracellular content of endogenous and exogenous substances in the brain (and other tissues). In neuroscience, it is often used to monitor dynamic changes in extracellular concentrations of neurotransmitters in mouse models in parallel with behavioral and pharmacological studies [1–3]. In order to detect imbalances between neurotransmitters within specific brain regions, such as the striatum, it requires simultaneous determination of different neurotransmitters and metabolites.

**Fig. 1:**
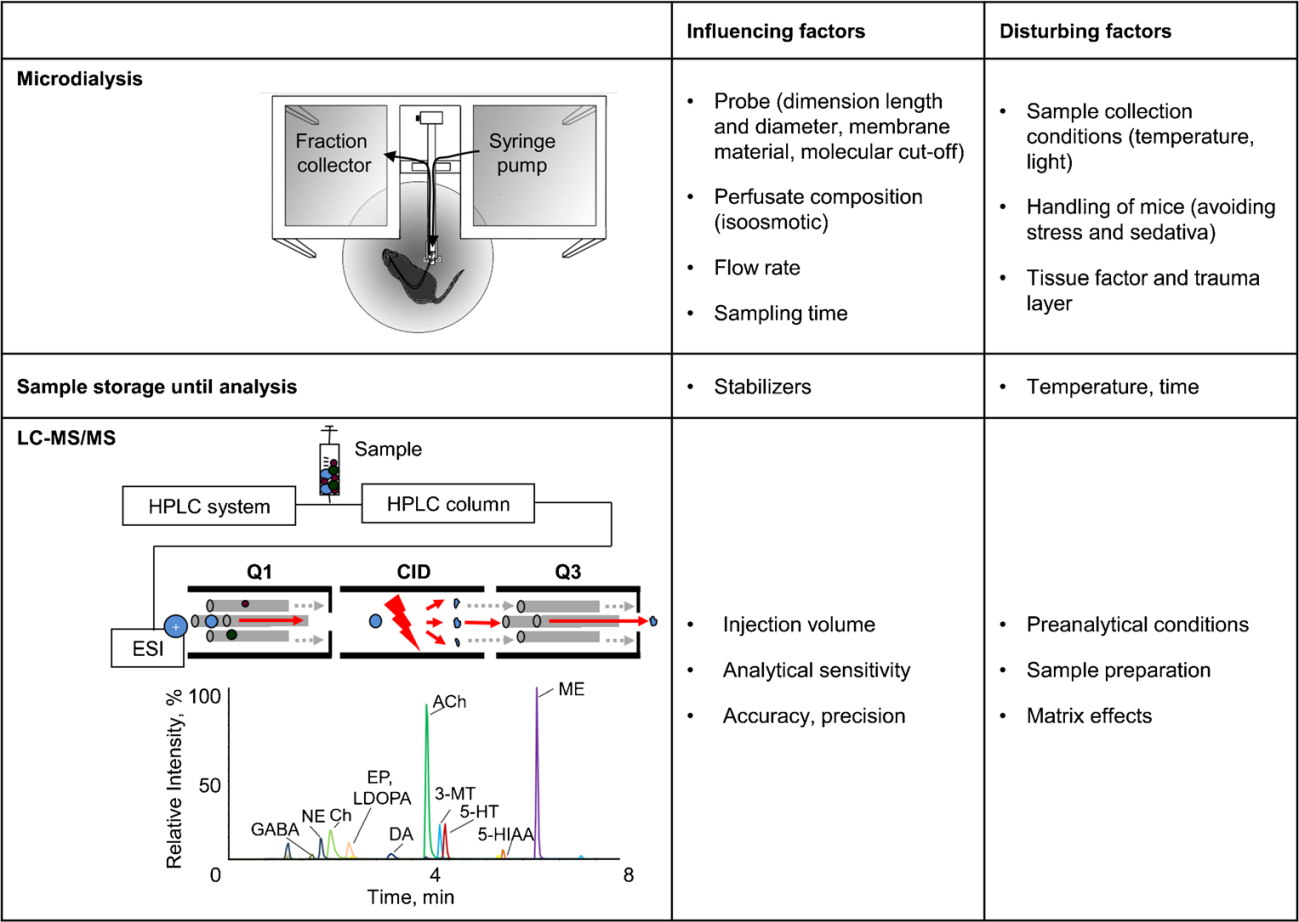
Influencing and disturbing factors in microdialysis technical setup with subsequent LC-MS/MS analysis

MD probes can be implanted into small brain structures in mice. The microdialysate contains all small- and middle-sized molecules that can diffuse inside the MD probe, depending on the properties of the membrane. The specific analyte determines the selection of the MD probe (membrane cutoff and probe dimension) as well as the composition of the perfusate (i.e., ion composition, stabilizer, or uptake blockers) that modifies the neurotransmitter content which has to be considered to determine basal differences or dynamic changes [2]. All these aspects as well as the instrumental MD setup, the perfusion flow rate, fraction collection time, and stimulation methods finally influence the development of the following quantitative analysis (Fig. 1). Furthermore, sample volumes of only a few microliters during defined time intervals are limiting factors for the subsequent quantitative analysis. Therefore, quantification requires a highly sensitive analytical method. This often hampers the quantification of different neurotransmitters to investigate changes of neurochemical imbalances. Most demanding requirements for the subsequent analysis are as follows: (I) high selectivity of the chromatographic separation; (II) high sensitivity to quantify concentrations in the expected range from picograms to nanograms; (III) short and gentle sample preparation, including avoidance of derivatizations due to the low sample volume and simultaneous determination of instable monoamines.

For years, high-performance liquid chromatography (HPLC) applications combined with fluorescence or electrochemical (EC) detection (ECD) was the common method for analyzing MD samples [1]. However, the main limitation of published HPLC-ECD methods, which detect easily oxidized analytes such as dopamine and serotonin, is the analysis of only a subset of neurotransmitters within one sample [1]. Acetylcholine (ACh) was detected mainly by fluorescence methods or indirectly by HPLC-ECD; however, best sensitivity is achieved by LC-MS/MS [4, 5]. Electrochemical detection requires further the application of an ACh esterase inhibitor in the perfusion medium, which might affect neuronal activity [6]. Also, previous studies applying ECD indicated an overestimation of neurotransmitter levels, probably caused by artifacts [7]. An advantage of LC-MS methods is that different groups of neurotransmitters can be quantified in one analytical run and therefore within the same sample [8–10] and the better discrimination between real analyte and interferences. With the advancing development of HPLC applications and MS technologies, new methods have been published for neurotransmitter quantification in the recent years. However, publications with focus on the combination of microdialysis and quantification of neurotransmitters by LC-MS/MS are limited and either included only few selected analytes in murine MD samples [11–13] or had disadvantageous analytical conditions such as long analysis times [14] or employed time-consuming sample preparation protocols [12, 14] including derivatization or sample volume reduction steps (freeze drying) [12, 15]. To the best of our knowledge, there are no multi-methods for analyzing basal neurotransmitter values in mice.

The aim of this study was to develop and validate a high-throughput LC-MS/MS method in combination with a MD setup for the sensitive analysis of basal values of an extensive neurotransmitter pattern in murine brain microdialysate, important for a follow-up study which includes multiple measurements in the striatum. Therefore, the following neurotransmitters were analyzed: serotonin (5-HT), 5-hydroxyindoleacetic acid (5-HIAA), melatonin (ME), dopamine (DA), levodopa (l-DOPA), 3-methoxytyramine (3-MT), norepinephrine (NE), epinephrine (EP), acetylcholine (ACh), choline (Ch), and γ-aminobutyric acid (GABA). A comprehensive in-house validation concept including stability studies was performed. The MD setup was validated including in vitro recovery studies. This method was used for the quantitative investigation of extracellular basal levels in murine striatum. However, this versatile method can also be applied for other brain areas and pharmaceutical intervention studies.

## Materials and methods

### Animals

Animal care was provided in accordance with the guidelines of the European Directive 2010/63/EU and the German Animal Welfare Agency. All experiments were approved by the Ethics Committee of the University of Leipzig under protocol number TVV31/16 (Landesdirektion Sachsen TVV31/16). Ten-month-old C57Bl/6J mice (*n* = 3) were used for microdialysis experiments. Mice were bred and group-housed in the institute’s facility on a 12-h light/12-h dark cycle in Makrolon cages (type III, not ventilated and open to environment) at 24 °C ± 2 °C with relative humidity of about 60%. Food (Altromin standard diet) and water were available ad libitum and material for nest building was provided. Stereotactic surgery was performed as described previously [16]. In brief, the microdialysis guide cannula was stereotactically implanted into the right murine striatum under 2.0% isoflurane anesthesia (CP-Pharma, Burgdorf, Germany) and 0.1% bupivacaine (Jenapharm, Jena, Germany) using the following coordinates in millimeter distance to the bregma and the skull surface according to the atlas of Franklin and Paxinos [17] in a stereotaxic frame (Stoelting, Wood Dale, IL, USA): anterioposterior + 0.8, mediolateral − 1.9, and dorsoventral 2.6. These coordinates were comparable to those used for previous experiments in mice [18]. The cannula was held in place with additional anchor screws and dental acrylic cement (Paladur^®^, Heraeus Kulzer, Germany) on the skull surface. To avoid obstruction, the guide cannula was equipped with a dummy cannula until the insertion of the microdialysis probe.

### Microdialysis procedure

After a post-surgery recovery period of at least 5 days, microdialysis experiments were performed in a freely moving system consisting of a plastic cylinder with a counterbalancing arm carrying a two-channel swivel (CMA 120, Carnegie Medicine). A microdialysis probe (CMA7, 1 mm, molecular cutoff 6 kDa; CMA Microdialysis AB, Kist, Sweden) was lowered gently through the guide cannula, in the process perfused at a rate of 0.5 μL/min with artificial cerebrospinal fluid (aCSF, final ion concentrations in mM: Na 150, K 3.0, Ca 1.4, Mg 0.8, P 1.0, Cl 155; Tocris Bioscience, Bristol, UK), which was interrupted during adaptation period. The mouse was put very carefully into the cage system for freely moving animals. Food and water gel were available ad libitum. After a 14–16-h recovery period, dialysate sampling started for 30-min intervals using a refrigerated fraction collector at 4 °C (CMA/170, Carnegie Medicine) in polypropylene tubes coated with 0.25 mM ascorbic acid/0.1 M perchloric acid. Six consecutive samples were collected for each animal. The aCSF inflow was driven by a CMA/100 microinjection pump (Carnegie Medicine) with a flow rate of 1.5 μL/min.

Depending on the MD in vitro recovery, the injection volume, and sensitivity of the subsequent LC-MS/MS analysis, the inflow rate and time intervals can be customized. Microdialysis was performed between 6:00 and 15:00 during the dark cycle under a minimum of light.

### Reagents and chemicals

The neurotransmitter standards and isotopically labeled internal standards were purchased from Sigma-Aldrich (Taufkirchen, Germany), d5-5HIAA from Sigma-Aldrich Co., Ltd. (Gillingham, GB), and d9-acetylcholine from Toronto Research Chemicals (Toronto, Canada). LC-MS pure methanol and acetonitrile were used from Merck KGaA LiChrosolv (Darmstadt, Germany) and VWR HiPerSolv (Darmstadt, Germany), respectively. Ultrapure water was obtained from a Milli-Q water purification system (Merck Millipore, Merck KGaA, Darmstadt, Germany). Formic acid was purchased from Sigma-Aldrich (Taufkirchen, Germany). Perchloric acid and ascorbic acid were obtained from Carl Roth (Karlsruhe, Germany). Polypropylene tubes, microdialysis sampling tubes, and glass vials were purchased from Sarstedt (Nümbrecht, Germany), Harvard Apparatus (Holliston, MA, USA) and Wicom (Heppenheim, Germany), respectively.

### Preparation of stock and working standards, calibrators, and quality control solutions

Standard stock solutions with concentrations of 1 mg/mL were prepared. Calibrators were prepared by serial dilution with water containing 0.25 mM ascorbic acid and 0.1 M perchloric acid. A total of 4 μL of calibrator solution was spiked to aCSF to final calibration concentrations of 0.025, 0.05, 0.25, 0.5, 0.75, 2.0, 10, 50, 75, and 100 ng/mL. Quality control samples with a final concentration of 0.6, 5, and 45 ng/mL aCSF were prepared as described for calibrators. Standards, calibrators, and quality control samples were aliquoted and stored at − 80 °C until analysis. The isotopically labeled standard was reconstituted in methanol to a concentration of 0.1 mg/mL or 1 mg/mL and diluted to the final concentration of 110 ng/mL in water containing 0.25 mM ascorbic acid and 0.1 M perchloric acid.

### Optimization of microdialysis conditions

As published by several research groups, the in vitro recovery depends on each individual microdialysate setup and is highly flow dependent [19, 20]. To determine the in vitro recovery for the microdialysate setup of the presented method, a neurotransmitter standard mixture of 15 ng/mL in aCSF was dialyzed at 21 °C with flow rates of 0.7, 1.0, 1.5, and 2.0 µL/min. Three samples per flow rate were collected.

### LC-MS/MS analysis

A Prominence UFLC system from Shimadzu (Duisburg, Germany), consisting of two low-pressure gradient pumps (LC-20 AD), an autosampler (SIL-20ACHT), a column oven (CTO-20 AC), and the controlling module (CMB-20A), coupled to a QTRAP LC-MS/MS 3200 from SCIEX (Framingham, MA, USA) was used. Analysis was performed with the security guard cartridge UHPLC Biphenyl RP-18e (3.0-mm ID columns) pre-column coupled to the analytical column Biphenyl RP-18e 100A 150 × 3.0 mm (2.6 μm), both obtained from Phenomenex (Aschaffenburg, Germany). The flow rate was 0.4 mL/min with solvent A, 0.1% formic acid in water; and solvent B, acetonitrile:methanol 1:1, v/v, with 0.1% formic acid. The isocratic gradient elution was as follows: 0–4.0 min from 0 to 70% B, 4.01–7.0 min 70% B, 7.01 0% B. During the first 1.0 min after sample injection, the eluent was switched to waste to avoid the ionization source from contamination due to aCSF matrix components. The column oven temperature was set to 30 °C. Positive electrospray ionization (ESI) mode was applied combined with multiple reaction monitoring (MRM) detection.

Quantitative analysis was carried out using the Analyst 1.5.1 software (SCIEX, Montreal, Canada).

### Sample preparation

Microdialysate samples were collected at 4 °C in sampling vials containing evaporated stabilizers (0.25 mM ascorbic acid and 0.1 M perchloric acid according to the collected sample volume). MD samples were directly stored at − 80 °C until LC-MS/MS analysis.

Forty microliters of MD sample and 4 μL internal standard solution were vortexed in autosampler vials with inserts and placed into the autosampler at 4 °C. Final concentration of internal standards in the sample was 10 ng/mL.

### Method validation

Potential matrix effects were investigated by comparison of the ratio of analyte to internal standard intensities in aCSF and water. Therefore, calibrators were prepared in aCSF and water. To evaluate co-elution of other substances and to detect contaminants, blank aCSF samples were analyzed in each analytical run.

The lower limit of detection (LLOD) was calculated from aCSF standard solutions according to the described sample preparation protocol using a signal-to-noise (*s*/*n*) ratio of 3:1. The lower limits of quantification (LLOQs) were stated as the *s*/*n* ratio of 10:1. Linearity of the neurotransmitters and related metabolites was tested up to 100 ng/mL.

The within-run and between-run imprecision was determined by preparing and analyzing each quality control sample 10 times and on 8 consecutive working days, respectively. The accuracy of the method was investigated by spike experiments. ACSF was spiked with standard solutions at appropriate concentration levels (quality control: 0.6, 5, 45 ng/mL).

Stability of analytes in processed samples stored in the autosampler at 4 °C until injection was assessed at analyte concentrations of 5 ng/mL. Spiked aCSF samples were aliquoted and measured 12 times over 18 h.

Stability of spiked aCSF samples (final concentration 5 ng/mL) was investigated for three freeze-thaw cycles. Samples were analyzed in triplicates for each experiment. Concentration levels after the first freeze-thaw cycle were used as reference for evaluation of freeze-thaw stability. Recovery rates from 80 up to 120% of the reference sample were evaluated as to be stable.

## Results and discussion

### Optimization of microdialysis conditions

Microdialysis represents one of the most powerful neurochemistry techniques. It is applied to gain information on extracellular neurotransmitter concentration in freely moving animals. The setup of this experimental approach requires surgical experience as well as consideration of several technical factors for the subsequent analyte quantification. For the final microdialysate setup, we evaluated the MD probe, the isoosmotic perfusate, the microdialysis flow rate, and the sampling time (Fig. 1).

We used a cuprophan membrane with a length of 1 mm and a molecular cutoff of 6 kDa as MD probe. The probe length was limited to the small size of mouse striatum. Artificial CSF was used as perfusate as it simulates the physiological extracellular fluid in the best possible manner. Sample collection was performed with a self-prepared stabilizer (ascorbic acid and perchloric acid) pre-coated MD tubes at 4 °C.

**Fig. 2:**
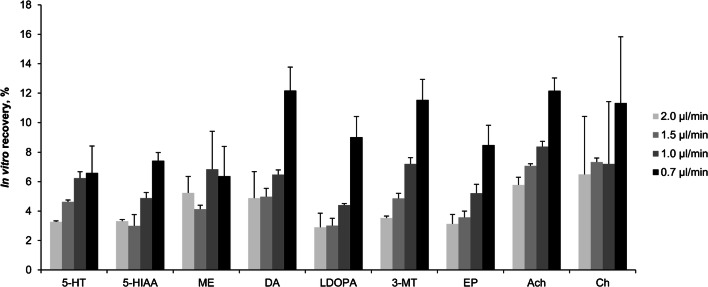
Microdialysis flow-dependent in vitro recovery, neurotransmitter standard mixture *c* = 15 ng/mL aCSF, *n* = 3 samples per flow rate, mean and SD for 5-HT (serotonin), 5-HIAA (5-hydroxyindoleacetic acid), ME (melatonin), DA (dopamine), l-DOPA (levodopa), 3-MT (3-methoxytyramine), EP (epinephrine), Ach (acetylcholine), Ch (choline), and GABA (γ-aminobutyric acid); NE (norepinephrine) was below the limit of quantification

The choice of the MD flow rate represents the most critical step for the following analyte quantification. The most suitable and conventionally applied microdialysate flow rate for neurotransmitter analysis varies between 0.3 and 2.0 μL/min with an in vitro analyte-dependent recovery between 5 and 20% [2, 20]. Reasons for these low recovery rates were the small dimension of the microdialysate probe combined with flow rates not allowing complete equilibrium between extracellular space and the interior of the probe. Very low MD flow rates below 0.2 μL/min increased the recovery to over 90%. However, we considered flow rates below 0.2 μL/min not suitable for our projects, as we aimed to apply the presented method to monitor relatively fast changes in the extracellular content and sampling time would be too long. In vitro recovery tests with our equipment and LC-MS/MS method for neurotransmitter and metabolite quantification resulted in the best recovery between 6 and 12% and a flow rate of 0.7 μL/min (Fig. 2). Nevertheless, we chose a flow rate of 1.5 μL/min and a sample collection time of 30 min with in vitro recoveries between 3 and 9% considering the aim to detect relatively fast changes.

### Optimization of chromatographic and tandem mass spectrometric conditions

**Fig. 3:**
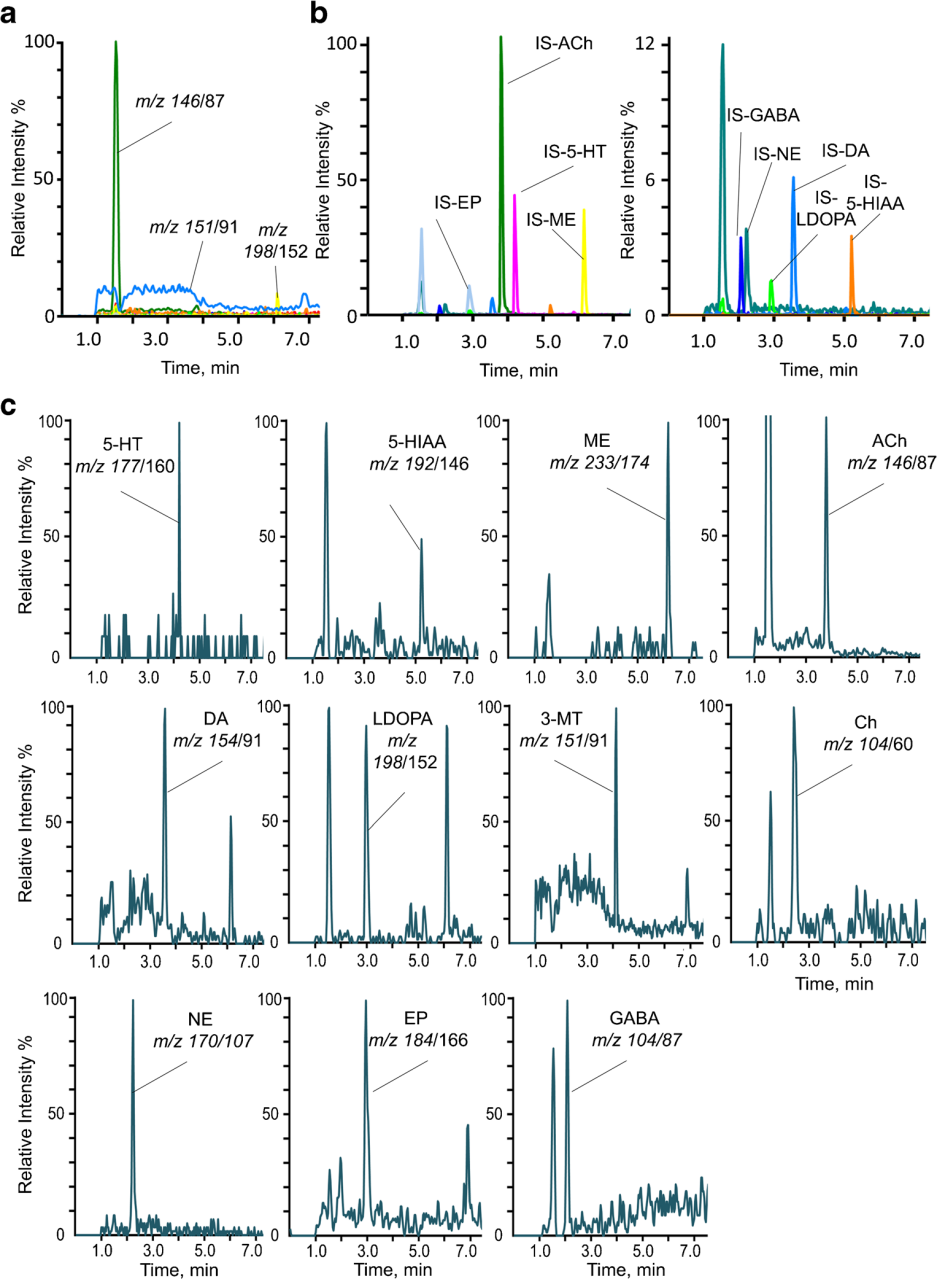
LC-MS/MS analysis of aCSF (a) chromatogram of a blank aCSF sample; *m/z* 146/87 (ACh, RT 3.84 min); *m/z* 151/91 (3-MT, RT 4.13 min); and *m/z* 198/152 (l-DOPA, RT 2.99 min); (b) chromatogram includes 9 internal standards (IS), *c* = 10 ng/mL; IS of l-DOPA and EP elute at the same time; (c) extracted mass transitions of each neurotransmitter in aCSF at the LLOQ; concentrations are between 0.025 and 2.0 ng/mL; injection volume was 25 μL

Best resolution and peak shape was achieved by employing a Kinetex biphenyl column and gradient elution over 4.5 min with mobile phases consisting of water with 0.1% formic acid (eluent A) and acetonitrile/methanol 50/50, v/v, with 0.1% formic acid (eluent B). These conditions retain the analytes of interest from the solvent front, which might lead to signal suppression effects during the ionization process due to the salt content in aCSF. The flow was diverted to waste during the first minute after injection to protect the mass spectrometer from contamination. A chromatogram under these optimized chromatographic conditions is presented in Fig. 3(b) for the internal standards and (c) for the extracted mass transitions of the analytes at the LLOQ.

Mass spectrometric parameters were optimized by separate infusion of each standard and internal standard solution into the TurboIon Spray^™^ Interface. The most intense product ions were used in the MRM mode to achieve maximum intensity. The following ESI source parameters were determined to receive highest signal intensities: ion source temperature was set to 650 °C, curtain gas at 30 psi and collision gas to medium; ionization voltage was set to 4500 eV; ion spray gas 1 at 40 psi and ion spray gas 2 at 65 psi. Mass transitions and analyte-specific mass spectrometric parameters are presented in Table 1.

**Table 1:** Mass spectral parameters used for the analysis of neurotransmitters, related metabolites, and their internal standard

	MRM (*m*/*z*)		DP (V)	EP (V)	CE (eV)	CXP (V)	RT (min)
Analyte	MS1	MS2					
5-HT	177.2	160.2	26	5	16	3	4.25
d_4_-5-HT	181.2	164.2	26	5	16	3	4.22
5-HIAA	192.0	146.2	30	10	21	2	5.25
d_5_-5-HIAA	197.0	150.2	37	6	20	4	5.23
ME	233.3	174.2	39	7	19	3	6.22
d_4_-ME	237.3	178.2	39	7	19	3	6.19
DA	154.3	91.3	20	10	31	3	3.60
d_4_-DA	158.3	95.3	20	10	31	3	3.55
l-DOPA	198.2	152.1	26	7	17	4	2.99
d_5_-l-DOPA	204.2	158.1	26	7	17	4	2.94
3-MT	151.2	91.2	52	7	29	3	4.13
NE	170.2	107.2	16	4	29	4	2.26
d_6_-NE	176.2	158.2	16	4	11	4	2.26
EP	184.2	166.2	27	4	15	3	2.98
d_6_-EP	190.2	172.2	27	4	15	3	2.93
ACh	146.1	87.1	26	7	21	3	3.84
d_9_-ACh	155.1	87.1	27	5	21	2	3.82
Ch	104.0	60.0	46	5	25	2	2.51
GABA	104.2	87.2	28	6	18	3	2.11
d_6_-GABA	110.2	93.2	28	6	18	3	2.10

*MRM* multiple reaction monitoring, *DP* declustering potential, *EP* extension potential, *CE* collision energy, *CXP* collision cell exit potential, *RT* retention time

Corresponding deuterated internal standards that enable to compensate for potential matrix effects were used for analyte quantification. Due to the structural similarity and the good recovery rates that were determined by using internal standard and matrix calibration curves, d4-dopamine and d9-acetylcholine were also used to quantify 3-methoxytyramine and choline, respectively, as surrogate IS. The injection of blank aCSF showed no signal intensities at analyte-specific retention times indicating no co-elution of other substances (Fig. 3).

### Method validation

Quantification of neurotransmitters and their metabolites was achieved by using calibration curves where the ratio of analyte peak area and internal standard peak area was plotted over the added concentration. Samples for the calibration points were prepared in aCSF. Each analytical run contained the following quality control samples three times (*c* = 0.6 ng/mL, 5 ng/mL, and 45 ng/mL).

Ranges of linearity for each analyte are presented in Table 2. Calibration curves were analyzed with a weighting of 1/*x*. Pearson’s coefficients of correlation were above 0.99 for all analytes. The limits of detection (LODs) with a signal-to-noise ratio of 3:1 and the lower limits of quantification (LLOQ) with a signal-to-noise ratio of 10:1 are included in Table 2. DA, 5-HT, ME, and ACh were lowest quantifiable with LODs below 0.05 ng/mL. LODs between 0.1 and 0.5 ng/mL were determined for 5-HIAA, LDOPA, 3-MT, EP, and Ch. NE and GABA were detectable at 0.75 ng/mL (Table 2).

**Table 2:** Limits of detection (LODs), lower limits of quantification (LLOQs), and linear ranges of neurotransmitters in aCSF

Analyte	LOD (ng/mL)	LLOQ (ng/mL)	Linear range (ng/mL)	*R*^2a^
5-HT	0.01	0.05	0.05–100	0.9996
5-HIAA	0.10	0.25	0.25–100	0.9992
ME	0.005	0.025	0.025–100	0.9998
DA	0.05	0.25	0.25–100	0.9994
l-DOPA	0.50	0.75	0.75–100	0.9996
3-MT	0.10	0.25	0.25–100	0.9990
NE	0.75	2.00	2.00–100	0.9966
EP	0.50	0.75	0.75–100	0.9982
ACh	0.025	0.05	0.05–100	0.9988
Ch	0.25	0.50	0.50–100	0.9992
GABA	0.75	1.00	1.00–100	0.9954

^a^*R*^2^ of the Pearson correlation

The intra- and inter-assay reproducibility for each analyte is presented in Tables 3 and 4, respectively. Intra-assay coefficients of variation varied between 4.6 and 21.2%. Due to the low intra-assay coefficients of variation, measurements of microdialysate samples could be performed in a single analysis. The mean inter-assay coefficient of variation for all analytes was 11.6%. As can be seen in Table 4, the accuracy ranged between 87 and 117% for all analytes. Hence, the data above show that neurotransmitters and metabolites included in this study can be quantified very reliably and accurately.

**Table 3:** Intra-assay coefficient of variation (CV) (*n* = 10) at three different concentrations across the range of linearity

Analyte	Mean *c*_1_^a^ (ng/mL)	CV	Mean *c*_2_^a^ (ng/mL)	CV	Mean *c*_3_^a^ (ng/mL)	CV
5-HT	0.44	8.8	4.74	7.3	56.29	7.6
5-HIAA	0.35	21.2	4.37	16.3	54.64	11.1
ME	0.45	13.5	4.84	10.6	51.27	9.3
DA	0.50	10.0	4.25	7.1	49.91	7.0
l-DOPA	-^b^		4.67	9.8	50.20	6.8
3-MT	0.60	13.1	4.51	9.0	50.06	13.7
NE	-^b^		4.49	19.1	57.72	13.1
EP	-^b^		5.04	7.1	52.96	6.2
ACh	0.34	4.6	3.85	7.1	43.17	6.3
Ch	0.54	15.5	5.61	9.3	56.27	6.7
GABA	- ^b^		5.18	14.1	53.81	10.8

^a^Spike concentrations: *c*_1_ = 0.5 ng/mL, *c*_2_ = 5 ng/mL, *c*_3_ = 55 ng/mL

^b^Below LLOQ

**Table 4:** Inter-assay coefficient of variation (CV) as well as the recovery rates (*n* = 8) at three different concentrations across the range of linearity

Analyte	Mean *c*_1_^a^ (ng/mL)	CV	Recovery (%)	Mean *c*_2_^a^ (ng/mL)	CV	Recovery (%)	Mean *c*_3_^a^ (ng/mL)	CV	Recovery (%)
5-HT	0.61	7.0	102	4.33	16	87	42.0	8.2	93
5-HIAA	0.62	11.1	103	4.38	8.9	88	46.1	7.6	93
ME	0.67	10.4	112	4.47	8.8	89	41.6	6.8	93
DA	0.61	9.6	101	4.58	6.8	92	44.1	4.0	98
l-DOPA	-^b^	-	-	4.76	11.5	95	45.1	10.0	100
3-MT	0.60	15.8	100	4.73	11.3	95	41.8	7.6	93
NE	-^b^	-	-	4.74	17.3	95	42.8	8.2	95
EP	-^b^	-	-	5.17	7.7	103	45.4	4.0	101
ACh	0.67	11.7	112	5.17	4.6	104	45.7	4.2	102
Ch	0.61	19.8	102	5.84	9.8	117	46.7	7.4	104
GABA	-^b^	-	-	4.99	17.3	100	43.5	7.7	95

^a^Spike concentrations: *c*_1_ = 0.6 ng/mL, *c*_2_ = 5 ng/mL, *c*_3_ = 45 ng/mL

^b^Below LLOQ

Only few LC-MS/MS methods have been published for the analysis of neurotransmitters in mouse brain microdialysate [7, 11, 21]. However, the major strength and superiority of the presented method is the analysis of as much as 11 analytes. This analytical setup enables the quantification of the serotonin and dopamine and related metabolites and additionally acetylcholine, choline, and GABA. Hence, a comprehensive picture of extracellular neurotransmitter levels can be provided by the implementation of our developed LC-MS/MS method.

Another advantage of the presented method is the short and gentle sample preparation procedure compared with time-consuming sample treatments including lyophilization and solid-phase extraction [15, 22], derivatization [12, 23], or centrifugation [24].

In comparison with other published LC-MS/MS methods, our analytical specification data shows a lower limit of detection for serotonin [24, 25] and one that is equivalent for 5-HIAA [26]. However, for dopamine, LLOQs of below 0.15 ng/mL are published [11, 24, 26–28] which might be explainable by either a different analytical setup [12, 29] or the development of a single analyte method for only dopamine quantification [27, 30]. In such methods, specifically optimized liquid chromatographic and mass spectrometric parameters can be applied. In multi-analyte methods, as presented here, a compromise for all analytes has to be found.

There are LC-MS/MS methods published for the quantification of ACh and Ch with much lower limits of detection than presented in our method [29, 31, 32]. However, the specific quantification of these two analytes is sufficient with our method with respect to the application of a fast and simple sample preparation. Furthermore, our results show that quantification of murine endogenous basal neurotransmitter and metabolite levels is possible with this analytical method.

We further investigated pre-analytical factors such as analyte stability in processed samples at 4 °C and over three freeze-thaw cycles. Our results show that aCSF samples spiked with 5 ng/mL of analytes stored in the autosampler at 4 °C are stable for 18 h (data not shown), which allows a batch size of all together 90 samples. Coefficients of variation between measurements during 18 h ranged from 3.98% for serotonin to 20.14% for norepinephrine. In this respect, our data for dopamine, norepinephrine, and serotonin are supported by Cannazza et al. [12].

Microdialysate samples retrieved for our studies were all deep frozen directly after sample collection and prior to tandem mass spectrometric analysis. Therefore, we analyzed changes in analyte concentrations over two and three further freeze-thaw cycles. Our results show that all analytes in spiked aCSF were stable for two freeze-thaw cycles (Table 5). After freezing aCSF a third time, only levels of levodopa seemed to change. Our data for dopamine, acetylcholine, and serotonin are supported by Cannazza et al. who also showed that these analytes remain stable for three freeze-thaw cycles [11].

**Table 5:** Analyte recovery (%) in aCSF samples spiked with 5 ng/mL after second and third freeze-thaw cycles (*n* = 3)

Freeze-thaw cycle	2	3
	Recovery (%)	Recovery (%)
5-HT	103	96
5-HIAA	98	87
ME	86	83
DA	104	108
l-DOPA	84	136
3-MT	106	98
NE	88	101
EP	98	106
ACh	97	98
Ch	113	98
GABA	105	108

Taken together, the following pre-analytical protocol for the analysis of microdialysis samples was established:

- Freeze collected microdialysate samples directly at − 80 °C
- Not more than two freeze-thaw cycles
- Thaw sample and prepare for mass spectrometric analysis
- Mass spectrometric analysis within 18 h while storing samples at 4 °C

### Application

**Fig. 4:**
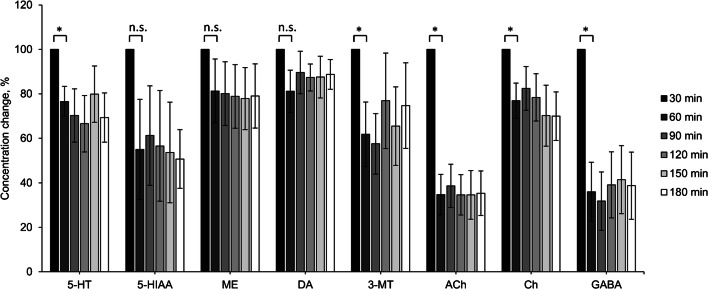
Striatal extracellular baseline concentration changes in percent over a time period of 3 h, after 18 h adaptation on inserted membrane, 5 days after stereotactic surgery implantation, *n* = 3 mice, mean ± SEM; l-DOPA, NE, and EP were below the LLOQ; *p < 0.05 using paired Student’s *t* test

Over a sampling time of 180 min with 6 fractions, the concentration changed for all analytes from the first to the second microdialysis sample (using paired Student’s *t* test), without any further significant changes (using a repeated measures ANOVA *p* < 0.05) in neurotransmitter concentration in the subsequent fractions 60 to 180 min (Fig. 4). A stable baseline is required for pharmacological studies. For this reason, we used the second striatal microdialysis sample; we detected the following mean basal levels of 3 mice: serotonin (mean 0.14 ng/mL), 5-HIAA (mean 6.79 ng/mL), melatonin (mean 0.28 ng/mL), dopamine (mean 0.48 ng/mL), 3-methoxytyramine (mean 0.46 ng/mL), acetylcholine (mean 0.26 ng/mL), choline (mean 13.2 ng/mL), and GABA (mean 1.20 ng/mL) (Table 6). As expected, neither epinephrine nor norepinephrine was detectable.

**Table 6:** Basal endogenous concentrations of detectable neurotransmitter and their related precursor and metabolites in the second microdialysate (MD) samples of murine striatum (*n* = 3)

Analyte	MD sample mouse 1, concentration (ng/mL)	MD sample mouse 2, concentration (ng/mL)	MD sample mouse 3, concentration (ng/mL)
5-HT	0.08	0.07	0.27
5-HIAA	3.79	8.48	8.11
ME	0.24	0.20	0.41
DA	0.54	0.50	0.40
3-MT	0.52	0.52	0.33
ACh	0.49	0.18	0.11
Ch	13.5	22.3	3.90
GABA	1.53	1.34	0.72

To the best of our knowledge, only very few studies are available that simultaneously measured absolute basal striatal extracellular neurotransmitter levels and related metabolites and none in mice. In addition, comparability across different studies is difficult as analyte levels depend (I) on the microdialysis setup, (II) on the detection system (MS/MS vs ECD), and (III) on animal condition (e.g., age, sex). As mentioned before, analyte levels highly depend on the microdialysate flow rate. Unfortunately, recovery rates were not considered in published data and no normalization was performed.

Basal striatal extracellular dopamine levels of 2.2 ± 0.4 nM (0.34 ± 0.06 ng/mL) [33] and of 6.1 ± 1.1 nM (0.93 ± 0.17 ng/mL) [34] and striatal acetylcholine levels of 0.008 ng/mL [35] have been reported. Nevertheless, all of the three mentioned studies applied HPLC-ECD for the quantification that did not allow the simultaneous determination of these analytes.

This is the first study providing absolute concentration levels of a comprehensive set of neurotransmitters and related metabolites quantified in mouse striatal microdialysate samples.

This method serves for further characterization of extracellular basal levels in several brain regions in different mouse models, to investigate neurotransmitter imbalances and to investigate the connection between the cholinergic, GABAergic, and dopaminergic system and abnormal neurological plasticity. It is therefore an essential basis for the combination with pharmacological intervention, optogenetic manipulation, or deep brain stimulation.

## Conclusion

A quantitative tandem mass spectrometric method was developed for the analysis of components of neurotransmitter pathways in murine microdialysate samples. This method enables for the first time the simultaneous quantification of metabolites of the dopamine and serotonin metabolism and furthermore choline, acetylcholine, and GABA in one sample.

A simple sample preparation procedure was applied. The validated LC-MS/MS method together with our pre-analytical protocol has been applied to the analysis of basal extracellular levels of biogene amines, neurotransmitter, and related metabolites in freely moving mice. This LC-MS/MS method can be applied for the investigation of basal levels and specific inter-neuronal stimulation or inhibition processes as well as neurotransmission.

## Abbreviations

ACh: Acetylcholine
aCSF: Artificial cerebrospinal fluid
Ch: Choline
DA: Dopamine
EC: Electrochemical
ECD: Electrochemical detection
ESI: Electrospray ionization
EP: Epinephrine
GABA: γ-Aminobutyric acid
5-HIAA: 5-Hydroxyindoleacetic acid
5-HT: Serotonin
LC-MS/MS: Liquid chromatography tandem mass spectrometry
l-DOPA: Levodopa
MD: Microdialysis
ME: Melatonin
3-MT: 3-Methoxytyramine
NE: Norepinephrine
NT: Neurotransmitter
